# Modification of the penicillin-binding-protein 3 as a source of resistance to broad-spectrum cephalosporins in *Escherichia coli*

**DOI:** 10.1093/jac/dkae020

**Published:** 2024-02-02

**Authors:** Samanta Freire, Jacqueline Findlay, Eva Gruner, Vera Bruderer, Patrice Nordmann, Laurent Poirel

**Affiliations:** Faculty of Science and Medicine, Medical and Molecular Microbiology, University of Fribourg, Fribourg, Switzerland; Faculty of Science and Medicine, Medical and Molecular Microbiology, University of Fribourg, Fribourg, Switzerland; Swiss National Reference Center for Emerging Antibiotic Resistance (NARA), University of Fribourg, Fribourg, Switzerland; Medica—Medizinische Laboratorien Dr F. Kaeppeli AG, Zürich, Switzerland; Medica—Medizinische Laboratorien Dr F. Kaeppeli AG, Zürich, Switzerland; Faculty of Science and Medicine, Medical and Molecular Microbiology, University of Fribourg, Fribourg, Switzerland; Swiss National Reference Center for Emerging Antibiotic Resistance (NARA), University of Fribourg, Fribourg, Switzerland; Faculty of Science and Medicine, Medical and Molecular Microbiology, University of Fribourg, Fribourg, Switzerland; Swiss National Reference Center for Emerging Antibiotic Resistance (NARA), University of Fribourg, Fribourg, Switzerland

Multidrug-resistant Gram-negative bacteria are one of the major threats to human health, largely due to the dissemination of some high-risk successful multidrug-resistant clones.^1^ Of particular concern is the resistance to ß-lactams, because those antibiotics are the most extensively used antimicrobial ones, accounting for half of all prescriptions in Europe.^2^ Resistance to broad-spectrum cephalosporins is of particular concern, as related infections require usage of carbapenems considered as last-resort antibiotics supposed to be preserved. Although the production of β-lactamases is the most common mechanism of resistance to broad-spectrum β-lactams in Gram-negative bacteria, and particularly in Enterobacterales, other mechanisms have also been reported including efflux, permeability changes and modification of PBP targets that contribute to this resistance.^3^

Our study was initiated by the isolation in March 2023 of an *Escherichia coli* strain recovered from a urine sample of an 86-year-old female patient hospitalized in Zürich, Switzerland, suffering from recurrent urinary tract infections. Corresponding antibiotic regimen were either cotrimoxazole or fosfomycin, but no ß-lactam has been used. Susceptibility testing by disc diffusion and broth microdilution showed that this isolate was resistant to broad-spectrum cephalosporins (ceftazidime, cefotaxime, cefepime) and to aztreonam, but remained susceptible to ceftazidime-avibactam and carbapenems, according to the EUCAST 2023 breakpoints.^4^ In addition, it was resistant to fluoroquinolones, nitrofurantoin, cotrimoxazole and fosfomycin.

Multilocus sequence typing performed as described in ref.^5^ identified that strain as belonging to ST1193, that has been reported as an emerging high-risk multidrug-resistant global clone.^1^ Resistance to broad-spectrum cephalosporins (ceftazidime, cefotaxime, cefepime) could not be explained by common mechanisms of resistance such as broad-spectrum ß-lactamases. Indeed, the Rapid ESBL NP test remained negative and PCR assays for all ESBL-encoding genes also remained negative.^6^ Likewise, PCR for all known AmpC β-lactamase encoding genes remained negative.

Whole-genome sequencing was therefore performed, using a short-read technology (MiSeq platform, Illumina, San Diego, CA, USA),^7^ and allowed the identification of mutations in *gyrA*/*parC* (*gyrA* S83L, D87N; *parC* S80I), responsible for fluroquinolone resistance,^1^ and an 8-bp insertion and subsequent disruption of *nfsA*, conferring resistance to nitrofurantoin.^8^ Regarding β-lactam resistance, the gene encoding the narrow-spectrum TEM-1 was the sole ß-lactamase gene identified, that explained the resistance to penicillins but not to the broad-spectrum cephalosporins.^9^ However, a four amino-acid insertion (YRVP) just after the amino-acid residue 333 (close to the β-lactam binding pocket) of the PBP-3 sequence was identified.

To investigate the role of this PBP-3 insertion in the resistance to cephalosporins, a wild-type PBP-3 was amplified from *E. coli* ATCC25922 reference strain using primers PBP3_F (5′-CCACGGAAAAGCTGCAAATG-3′) and PBP3_R (5′-CATCGGTCGCCTCATCTTTC-3′) and cloned into plasmid pCR-Blunt II-TOPO,^7^ then the corresponding recombinant plasmid transformed into the *E. coli* clinical isolate. Hence, recovery of susceptibility to broad-spectrum cephalosporins was observed in the complemented strain (Table 1).

**Table 1.** MICs of β-lactams and non-β-lactams for the clinical strain, clinical strain with an empty vector (pTOPO) and clinical strain complemented with a recombinant plasmid possessing a gene encoding a non-mutated PBP-3 (pPBP-3)

Mic (µg/mL)	N3940	N3940 (PTOPO)	N3940 (PPBP-3)
**TIC**	>256	>256	>256
**AMX**	>256	>256	>256
**TEM**	128	128	64
**CAZ**	8	8	1
**CZA**	8	8	2
**CTX**	4	4	1
**FEP**	4	4	1
**FOX**	128	128	128
**FDC**	0.25	0.25	0.25
**ATM**	8	8	1
**AZA**	8	8	1
**ETP**	<0.06	<0.06	<0.06
**MEM**	<0.06	<0.06	<0.06
**IMI**	<0.06	<0.06	<0.06
**NTF**	>256	—	—
**CIP**	64	—	—
**KAN**	1	—	—

TIC, ticarcillin; AMX, amoxicillin; TEM, temocillin; CAZ, ceftazidime; CTX, cefotaxime; FEP, cefepime; FOX, cefoxitin; FDC, cefiderocol; CZA, ceftazidime-avibactam; AZA, aztreonam-avibactam; ATM, aztreonam; ETP, ertapenem; MEM, meropenem; IMI, imipenem; NTF, nitrofurantoin; CIP, ciprofloxacin; KAN, kanamycin.

—, not evaluated.

A similar PBP-3 modification has been recently evidenced among NDM-1 and NDM-5 *E. coli* isolates already been reported,^1,10^ with either YRIN or YRIK 4-amino-acid insertions also identified after position 333. It was shown that these PBP-3 modifications were responsible for resistance to aztreonam and consequently also to the aztreonam-avibactam combination, which is a significant concern as those isolates were co-producing NDM-like carbapenemases along with AmpC-type CMY ß-lactamases, resulting in high-level resistance to all ß-lactams including carbapenems.

Overall, we showed that the YRVP insertion in the PBP-3 was involved in the decreased susceptibility to cephalosporins, such as ceftazidime, cefotaxime, cefepime and aztreonam, a phenotype observed without production of any acquired broad-spectrum ß-lactamase. This implies that recovery of an increased susceptibility cannot be expected when considering combinations made of ß-lactam/ß-lactamase inhibitor such as ceftazidime-avibactam, as highlighted here with an MIC at 8 mg/L either for ceftazidime and for ceftazidime-avibactam (a value being at the exact EUCAST breakpoint for the latter, whereas that of ceftazidime is actually at 1 mg/L). Zhang *et al*. previously highlighted that PBP-3 modifications so far described did not confer resistance to CAZ-AVI but recommended accurate monitoring.^11^ Our observations are fully in accordance with this warning. In addition, the isolate investigated here belonged to the ST1193, recently considered to be an emerging high-risk multidrug-resistant global clone responsible for urinary tract and bloodstream infections.^1^
